# Association between Glycemic Control and Clinic Attendance in Emerging Adults with Type 1 Diabetes: A Tertiary Center Experience

**DOI:** 10.1155/2018/9572817

**Published:** 2018-07-09

**Authors:** Eldad Fisher, Liora Lazar, Shlomit Shalitin, Michal Yackobovitch-Gavan, Liat de Vries, Tal Oron, Ariel Tenenbaum, Moshe Phillip, Yael Lebenthal

**Affiliations:** ^1^Sackler Faculty of Medicine, Tel Aviv University, Tel Aviv 69978, Israel; ^2^The Jesse Z and Sara Lea Shafer Institute for Endocrinology and Diabetes, National Center for Childhood Diabetes, Schneider Children's Medical Center of Israel, Petah Tikva 49202, Israel

## Abstract

**Aims:**

The transition of emerging adults with type 1 diabetes (T1D) from pediatric diabetes clinics to adult clinics between 18 and 21 years of age could result in decreased clinic attendance and thus worsen glycemic control. Our institutional policy offering surveillance till age 30 enabled us to evaluate clinic attendance without the confounding effect of transition. Our aim was to determine the association between glycemic control (HbA1c) and attendance rate.

**Methods:**

The medical records of 261 (54% males) young adult T1D patients (median age 22.9 years) were reviewed. Patients were stratified according to the attainment/nonattainment of glycemic targets (HbA1c ≤ 7% versus HbA1c > 7% (53 mmol/mol)). The attendance rate was calculated as the number of clinic visits/number of scheduled appointments.

**Results:**

Median annual number of scheduled visits was 3 (3, 4); attendance rate was 75% (53.6%, 100%). Seventy-four (28.4%) patients attained glycemic targets (median HbA1c 6.5% (48 mmol/mol) (6.3%, 6.8% (45.51 mmol/mol)); 187 (71.6%) patients had a median HbA1c of 7.8% (62 mmol/mol) (7.4%, 8.4% (57.68 mmol/mol)). The attainment of the treatment target was more prevalent in older patients (*P* = 0.006), in male patients (*P* = 0.007), and in patients with higher education (*P* = 0.017). Higher attendance rate (*β* (2.483), *P* < 0.001) and male gender (*β* (0.746), *P* = 0.015) were associated with better metabolic control.

**Conclusions:**

In emerging adults with T1D during the ongoing stable phase of diabetes management, higher attendance rate, rather than absolute number of clinic visits, was associated with the attainment of glycemic targets.

## 1. Introduction

The Diabetes Control and Complications Trial (DCCT), based on a regimen of monthly clinic visits with a multidisciplinary team, clearly demonstrated the positive impact of intensive diabetes therapy on glycemic control [1]. Since then, it has come to be widely accepted that the more practical regimen of 3-4 annual visits, as advocated by the American Diabetes Association (ADA), constitutes a satisfactory therapeutic regimen for adults with T1D [2, 3]. The routine clinical practice followed for young adults in our tertiary center for diabetes has been quarterly clinical visits during which HbA1c is evaluated and diabetes management is reassessed. Yet, diabetologists can recommend a fewer or greater number of visits per their clinical judgement [3]. T1D patients who have unsatisfactory glycemic control are invited more frequently in an attempt to promote adherence to treatment regimen.

Emerging adulthood (defined as age 18–30 years) is a period when patients are expected to assume greater responsibility for their diabetes care, while parents reduce their involvement in management of the disease [4]. Current guidelines endorse the transition of T1D patients from pediatric diabetes clinics to adult clinics between 18 and 21 years of age [5]. Several groups have reported concern that the transition of emerging adults at this young age could result in decreased clinic attendance and thus worsening glycemic control [5–9]. Our institutional policy in the past two decades has been to offer continuing surveillance by our multidisciplinary team until the much later age of 30 years. This practice has enabled us to determine whether clinic attendance affects glycemic control in emerging adults with T1D without the confounding effect of transition. Our hypothesis was that well-controlled type 1 diabetes-emerging adult patients could attend the clinic less frequently than 4 times a year. The objective of the present study was to determine the association between glycemic control (HbA1c) and clinic attendance rate during the period of emerging adulthood and to assess the influence of sociodemographic and diabetes-related parameters on clinic attendance.

## 2. Patients and Methods

### 2.1. Patients

A survey of all patients attending the National Center for Childhood Diabetes at the Schneider Children's Medical Center of Israel (SCMCI) between January 1st, 2011 and December 31st, 2011, yielded 397 emerging adult T1D patients aged 18 to 30 years. Of these, 261 patients fulfilled the following inclusion criteria: duration of T1D for at least one year prior to data retrieval with follow-up during 2012 (indicating continued surveillance). Excluded from the study were 136 patients: 45 discontinued follow-up in our center, 33 required more intensive diabetes surveillance, with >4 clinic visits, 21 participated in an intervention study, 21 changed mode of therapy from multiple daily injections (MDI) to continuous subcutaneous insulin infusion (CSII) and participated in a 3-day education session, and 16 had a recent onset of diabetes. During the studied period of surveillance, the population of emerging adults was categorized as follows: 65.7% (261/397)—ongoing stable phase of diabetes management (defined as the period beyond initial diagnosis and early diabetes education—the study cohort); 23% (91/397)—more intensive diabetes management (recent onset of T1D, more frequent clinic visits due to participation in an intervention study or changing mode of therapy); and 11.3% (45/397)—discontinued follow-up in our center.

The study protocol was approved by our Institutional Review Board. Informed consent was waivered by our Institutional Review Board.

### 2.2. Methods

This was a retrospective study of a cohort of emerging adult T1D patients followed at our tertiary center for diabetes. Retrieved from medical files were sociodemographic parameters (age, gender, ethnicity, region of residence, distance from clinic, and employment status) and diabetes-related parameters (age at diagnosis of T1D, number of clinic visits, number of appointments made, HbA1c levels, mode of therapy, whether MDI or CSII, episodes of DKA, severe hypoglycemia, and diabetes-related hospitalizations).

Attendance (kept appointment) rate was calculated as the number of clinic visits/number of scheduled appointments. Capillary HbA1c was measured by an automated immunochemical technique (DCA 2000; Bayer Diagnostics Inc., Tarrytown, NY, USA; 95% confidence interval (CI) 4.3–5.7%). HbA1c was routinely tested at each visit. Median HbA1c was calculated for each patient using the available HbA1c values during the one-year study period. Target HbA1c was defined according to ADA guidelines in emerging adults as ≤7% (53 mmol/mol) [10]. DKA was defined as an episode of hyperglycemia and ketoacidosis leading to an emergency department visit and/or hospital admission. Severe hypoglycemia was defined as a hypoglycemic episode associated with severe cognitive impairment requiring external assistance for recovery and treated at home with glucagon, severe cognitive impairment requiring the assistance of another person or requiring assistance in an emergency department visit, or severe cognitive impairment requiring hospital admission. Data on DKA and/or severe hypoglycemia occurring during the preceding months were reported and recorded at each visit.

### 2.3. Statistical Analysis

The data were analyzed using SPSS statistical software (release 23.0; SPSS Inc., Chicago, IL). All statistical tests were performed as two sided. The Kolmogorov-Smirnov *z* test was performed to test the null hypothesis that the variable has a normal distribution. Data are expressed as median and interquartile range (IQR) for skewed distribution. HbA1c was stratified into 2 categories (≤7% (53 mmol/mol), >7% (53 mmol/mol)) and comparisons of demographic and clinical parameters were performed. Pearson's chi-square test was used for analysis of between-group differences in discrete variables. Independent-samples Mann–Whitney *U* test was used to compare between groups for continuous variables (all had a skewed distribution). Stepwise multivariate logistic regression analysis (backward LR) was used to define factors potentially affecting target HbA1c. A *P* value of ≤ 0.05 was considered significant.

## 3. Results

The cohort of 261 emerging adults (54.4% males) had a median age of 22.9 years (IQR 20.7, 25.9). Median age at diagnosis of T1D was 11.8 (IQR 10.4, 12.9) with a median diabetes duration of 11.1 years (IQR 7.8, 15.5). Sociodemographic characteristics of the entire cohort revealed that only a minority were unemployed (3.8%), and only a few (1.1%) did not graduate high school. Most resided in an urban area (78.9%), with a median distance from the clinic of 20 km (IQR 10, 41).

In the entire cohort, the median number of annual scheduled visits was 3 (IQR 3, 4) and the attendance rate was 75% (IQR 53.6%, 100%). During this period, treatment modality was MDI in 58.6% of the emerging adults and CSII in 41.4%. Autoimmune comorbidities were found in 13.2% of the cohort and microvascular complications in 7.3%. Acute diabetes-related complications—severe hypoglycemic events, DKA episodes, and diabetes-related hospitalizations—were documented in 1.5%, 1.1%, and 2.3% of the patients, respectively.

Median HbA1c of the studied cohort was 7.5% (59 mmol/mol) (IQR (6.9%, 8.1%) (52, 65 mmol/mol)). Seventy-four (28.4%) of the patients attained treatment targets (HbA1c ≤ 7%) with a median HbA1c of 6.5% (48 mmol/mol) (IQR (6.3%, 6.8%) (45, 51 mmol/mol)); 187 (71.6%) of the patients had a median HbA1c of 7.8% (62 mmol/mol) (IQR (7.4%, 8.4%) (57, 68 mmol/mol)). Patients who attained treatment targets had a significantly lower number of scheduled visits (median (IQR): 2 (2, 3) versus 3 (2, 3), *P* < 0.001) but a higher attendance rate (median (IQR): 100 (67, 100) versus 75 (50, 100), *P* = 0.020).

Sociodemographic characteristics (Table 1) showed that the attainment of the treatment target was more prevalent in older patients (*P* = 0.006), in male patients (*P* = 0.007), and in patients with higher education (*P* = 0.017). Ethnicity, employment status, and distance from the diabetes clinic did not significantly affect the attainment of glycemic control.

Diabetes-related characteristics of the studied cohort according to the attainment of treatment targets is presented in Table 2. Median age at diagnosis of diabetes was significantly older in patients attaining treatment targets (13.3 versus 11.5 years, *P* = 0.020), with no significant difference in diabetes duration and autoimmune comorbidities. Treatment modality was similar among patients with HbA1c ≤ 7% and those with >7% (CSII in 44.6% versus 40.1%, *P* = 0.600). No significant differences were found between groups in regard to diabetes-related complications per 100 patient-years (hypoglycemic events: 2.7 versus 1.1, *P* = 0.333, diabetic ketoacidosis: 1.4 versus 1.1, *P* = 0.847, diabetes-related hospitalizations: 2.7 versus 2.2, *P* = 0.789, and microvascular complications: 0.8 versus 0.6, *P* = 0.454).

Predictors for metabolic control in emerging adults are presented in Table 3. The final logistic regression model showed that a better kept attendance rate (*β* (2.483), *P* < 0.001) and male gender (*β* (0.746), *P* = 0.015) were associated with a better metabolic control. This model explains 11.4% of the variance in HbA1c levels (*R*^2^ = 0.114).

## 4. Discussion

Our institutional policy offering continuing surveillance of young adults up to age 30 within the framework of our pediatric services enabled us to evaluate the importance of clinic attendance as recommended by the multidisciplinary diabetes team without the confounding effect of transition. The present study revealed that in emerging adult patients with T1D during the ongoing stable phase of diabetes management, a higher kept appointment rate was associated with the attainment of better glycemic control.

During the period of emerging adulthood, T1D patients are at increased risk of a declining frequency of clinic visits and even possible disengagement from health care surveillance [6–8, 11, 12]. Although our institutional policy encourages all emerging adults to remain under our surveillance, 11% of the patients in this cohort discontinued their follow-up in our center. Discontinuation of follow-up in our institute cannot be attributed to the financial burden of healthcare since in Israel healthcare service is mandated for all residents via the endorsement of the Health Insurance Law. Diabetes patients benefit from comprehensive, publicly funded, and administered care which includes doctor visits, diagnostic and laboratory services, hospitalization, and subsidized prescription medications.

Our emerging adult cohort who continued their follow-up in our clinic attended an average of 75% of scheduled visits, an outcome better than previously reported by others [11–13]. This relatively high kept appointment rate may stem from an ascertainment bias as motivated young adults are more likely to decide to remain under our care, while those less motivated are more apt to leave our facility.

Previous studies have reported that metabolic control and diabetes-related outcomes were associated with clinic attendance: patients having frequent contact with the diabetes clinic received continued diabetes education and had a better course and outcome than those with poor or sporadic attendance [9, 12, 13]. Dyer et al. reported that nonattendance was a significant problem in young adult T1D patients; still, glycemic control was significantly improved in those who attended at least three-quarters of their appointments [11]. Interestingly, we found that in young adulthood higher clinic attendance rate was associated with better glycemic control. Thus, attendance rate can serve as a marker of compliance with treatment suggesting that well-controlled patients with a high level of diabetes competency could be scheduled for fewer clinic visits.

The metabolic control of our emerging adult cohort during the ongoing stable phase of diabetes management, as expressed by the median HbA1c of 7.5%, was near the recommended target and 28% attained the treatment target of 7% (ADA, ISPAD guidelines). Our findings are in agreement with reports based on individuals with type 1 diabetes participating in the T1D Exchange Clinic Registry and international diabetes registries [14–25]. The incidence of severe hypoglycemia, DKA episodes, and diabetes-related hospitalizations was lower than that reported in other population-based studies [14–25]. These findings are consistent with those of our previous report of a significantly lower percentage of acute diabetes-related complications in young adulthood [26], although it must be noted that the frequencies of severe hypoglycemia, DKA, and hospitalizations may be underreported.

Our data demonstrated that emerging adult males had a better likelihood to attain metabolic treatment targets than females. Gender differences in young adults with type 1 diabetes have been previously investigated [14–17]. In line with previous reports from large databases, HbA1c levels were consistently lower in males than in females and a higher percentage of male subjects attained treatment targets [14–17].

The employment status of our young adult patients did not influence adherence to scheduled appointments. This is in line with the study by Dyer et al., which found that attendance rates were not associated with employment status [11]. In Israel, patients do not pay out of pocket for clinic visits and paid absence from work for medical reasons is justified. Therefore, not attending scheduled appointments cannot be attributed to the financial burden of the disease. In addition, the geographic distance from the clinic had no impact on clinic attendance. It should be noted that although T1D patients are referred to our tertiary care center from all over the country, most of our population of emerging adults reside in close proximity to the clinic (~20 km). Moreover, in general most of our health care services are quite easily available, which may not be possible in other countries with a wider geographical population distribution.

The major strength of this study is the large cohort of T1D patients receiving a similar standard of clinical care and uniform HbA1c determination in a single tertiary care center. It does, however, have several limitations. The major limitation is that this is a single center clinical observational study in the unique context of continued diabetes care and surveillance of patients until age 30 years of age. Due to its retrospective nature, we could not use questionnaires pertaining to diabetes competency levels and adherence to the diabetes regimen. The reasons for canceling scheduled appointments are not routinely documented in the patient's file. Moreover, the fact that our multidisciplinary team is available 24 hours a day for consultation could have had an impact on glycemic control and may also have resulted in lower attendance. Although our tertiary care center serves all sectors of the Israeli population, including patients of various ethnic origins and social-economic status from both urban and rural areas, our findings may not be applicable for other clinics in Israel or abroad.

In conclusion, our findings suggest that in emerging adult patients with T1D during the ongoing stable phase of diabetes management, a higher attendance rate, rather than the absolute number of clinic visits, was associated with the attainment of better glycemic control. Our institutional policy endorses quarterly visits in order to obtain maximum benefit from the multidisciplinary team of specialists, thus reinforcing good self-care practice. Still, there may be a select group of patients able to maintain good metabolic control with less frequent visits.

## Figures and Tables

**Table 1:** Sociodemographic characteristics of emerging adults with type 1 diabetes stratified by the attainment of treatment targets.

HbA1c, % (mmol/mol)	≤7 (53)	>7 (53)	*P*
*Patients*, *n(%)*	74 (28.4)	187 (71.6)	
*Current age, median (IQR), years*	23.9 (21.4, 27.1)	22.5 (20.5, 25.2)	0.006
*Gender*, *n(%)*			
Male gender	50/142 (35.2)	92/142 (64.8)	0.007
Female gender	24/119 (20.2)	95/119 (79.8)	
*Ethnicity*, *n(%)*			
Jewish	71/245 (29.0)	174/245 (71.0)	0.191
Arab	2/15 (13.3)	13/15 (86.7)	
*Employment*, *n(%)*			
Unemployed	1 (1.4)	9 (4.8)	0.129
Military/national service	8 (10.8)	34 (18.2)	
Student	30 (40.5)	79 (42.2)	
Employed	35 (47.3)	65 (34.8)	
*Adult education*, *n(%)*			
Elementary	1 (1.4)	2 (1.1)	0.017
High school	49 (66.2)	154 (82.8)	
University	24 (32.4)	31 (16.6)	
*Distance from clinic, median (IQR), km*	15.5 (8, 37)	20 (10, 41)	0.132

Data presented as median and interquartile range (IQR), number (*n*), and percentage (%). HbA1c, glycated hemoglobin. *P* ≤ 0.05 was considered significant.

**Table 2:** Comparison of diabetes-related characteristics of emerging adults with type 1 diabetes during the studied year stratified by the attainment of treatment targets.

HbA1c, % (mmol/mol)	≤7 (53)	>7 (53)	*P*
Patients, *n* (%)	74	187	
Age at diagnosis, median (IQR), years	13.3 (10.1, 16.3)	11.5 (8.8, 14.4)	0.020
Duration of diabetes, median (IQR), years	11.4 (7.1, 16.1)	11.0 (8.2, 15.3)	0.877
Autoimmune comorbidities, *n* (%)	13 (17.6)	33 (17.6)	1
Current treatment modality, *n* (%)			
Multiple daily injections	41 (55.4)	112 (59.9)	0.600
Insulin pump	33 (44.6)	75 (40.1)	
Annual number of scheduled visits, median (IQR)	3 (2, 4)	4 (3, 5)	<0.001
Annual number of clinic visits, median (IQR)	2 (2, 3)	3 (2, 3)	0.043
Attendance rate, median (IQR), percentage	100 (67, 100)	75 (50, 100)	0.020
Presence of hypoglycemic events, per 100 patient-years	2.7	1.1	0.333
Presence of diabetic ketoacidosis, per 100 patient-years	1.4	1.1	0.847
Presence of diabetes-related hospitalizations, per 100 patient-years	2.7	2.2	0.789
Microvascular complications, per 100 patient-years	0.83	0.58	0.454

Data are presented as median and interquartile range (IQR) unless otherwise indicated. HbA1c, glycated hemoglobin; *n*, number; %, percentage. *P* ≤ 0.05 was considered significant.

**Table 3:** Final logistic regression model for variables associated with the attainment of target HbA1c.

	*β* (SE)	*P*	OR	95% CI for OR
*Intercept*	−4.513 (1.263)	<0.001		
*Attendance rate*	2.483 (0.685)	<0.001	11.98	3.13, 45.84
*Clinic visits*	−0.523 (0.177)	0.003	0.59	0.42, 0.84
*Gender (male)*	0.746 (0.307)	0.015	2.11	1.16, 3.85
*Current age*	0.080 (0.042)	0.056	1.08	1.0, 1.18
*Diagnosis age*	0.055 (0.032)	0.090	1.06	0.99, 1.13

All significant variables from the univariate analyses were included in the logistic regression model (see Tables 1 and 2): gender, current age, age at diagnosis of diabetes, attendance rate, and number of clinic visits. *β*, variable coefficient; SE, standard error; OR, odds ratio; CI, confidence interval.

## Data Availability

The data used to support the findings of this study are available from the corresponding author upon request.
